# Vaginal Cuff Dehiscence Following Remote Hysterectomy: An Unusual Case of Bowel Evisceration

**DOI:** 10.1155/2024/2074381

**Published:** 2024-04-24

**Authors:** Harsh Desai, Alexander Canales

**Affiliations:** Department of Surgery, Division of Trauma, Surgical Critical Care and Acute Care Surgery, Allegheny Health Network, Forbes Hospital, Monroeville, Pennsylvania 15146, USA

## Abstract

Hysterectomy is one of the most common gynecologic procedures performed worldwide. Vaginal cuff dehiscence with resultant bowel evisceration, while rare, is one of the most serious complications of the procedure. We present the case of a 79-year-old female with vaginal cuff dehiscence several decades after hysterectomy. The patient had no significant antecedent symptoms until she experienced bowel evisceration during Valsalva. She underwent a successful reduction of bowel contents with limited resection and transvaginal cuff repair. This case highlights the risk of vaginal cuff dehiscence even decades following the procedure.

## 1. Introduction

Aside from cesarean section, hysterectomy is one of the most common procedures performed in the field of gynecology. Vaginal cuff dehiscence, either full thickness or partial, has a reported incidence of 0.14% to 4.1%. Even more rare is evisceration of intraperitoneal contents, reported to have an incidence of 0.032% to 1.2% [1, 2]. The distal ileum has been noted as the most common eviscerated organ, though the colon, fallopian tubes, and omentum have also been noted [3]. Numerous studies have reported an increased risk of vaginal cuff dehiscence with laparoscopic and robotic hysterectomy as opposed to total abdominal hysterectomy and total vaginal hysterectomy [4].

Considering the rare incidence of evisceration, most literature has been in the form of case series, retrospective reviews, and case reports. In addition to the type of hysterectomy, several other risk factors have been linked to vaginal cuff dehiscence categorized broadly into surgical factors, increased intra-abdominal pressure, and ineffective wound healing [1]. Surgical factors studied include insufficient bites during tissue closure and excessive electrocautery use resulting in tissue attenuation. Factors increasing intra-abdominal pressure include constipation, coitus in the healing period, heavy lifting, or repeated vigorous Valsalva (such as with chronic cough). Finally, ineffective wound healing has been linked to age, chemotherapy and malignancy, chronic steroid use, chronic immunosuppressive conditions such as poorly controlled diabetes, and postmenopausal state [1, 4]. Here, we describe a case of vaginal dehiscence with evisceration remote from index hysterectomy.

## 2. Case Report

**Figure 1:**
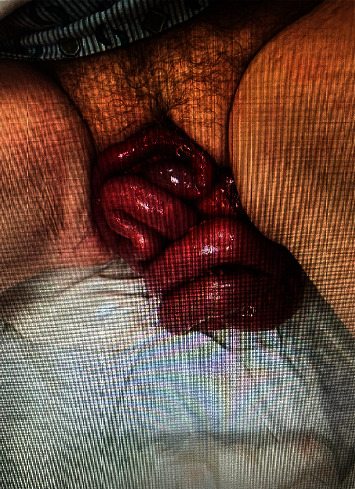
Vaginal cuff dehiscence with bowel evisceration.

The patient is a 79-year-old female with a past medical history of hypertension and anxiety who presented to the emergency department with a complaint of bowel evisceration. The patient stated that she had been in the lavatory attempting a bowel movement at which time she felt vaginal fullness and then a sudden expulsion of bowel. The patient's spouse called paramedics and she was transported in hemodynamically stable condition to the emergency department. Acute Care Surgery was emergently consulted for evaluation. During the interview, the patient denied symptoms of a vaginal bulge or increased pressure with bowel movements prior to the evisceration. She further denied bowel or bladder dysfunction. She indicated transvaginal hysterectomy approximately 40 years prior with no further gynecologic procedures of note since. On social history, she denied cigarette use now or in the past and indicated occasional alcohol use. On exam, the patient was hemodynamically stable and nonobese (BMI 24) with evisceration of several loops of the small intestine and a loop of colon noted (Figure 1).

The patient remained hemodynamically stable and in full faculty throughout the evaluation in the emergency department. After obtaining informed consent, the patient was taken to the operating room for exploration within approximately 2 hours of presentation. The procedure began with a lower midline laparotomy incision with the patient in the lithotomy position. Inspection of the abdominal contents noted evisceration starting at the point of the distal jejunum along with a loop of the sigmoid colon. At this juncture, gynecology was called to assist with the reduction of contents through the vaginal canal, and working in tandem successful reduction was achieved. On external inspection, the vaginal cuff appeared edematous, but otherwise healthy. A sutured, transvaginal closure was conducted of the cuff by gynecology. Intra-abdominally, the bowel was evaluated, and the eviscerated portion was noted to be somewhat hemorrhagic with only a small loop of ileum noted to be ischemic. This loop was noted to be fused to the vaginal cuff. As such, the loop was resected with a cuff of fused bowel wall left in place. An AbThera vacuum dressing (3M, 2024) was placed for temporary abdominal closure during this index operation, and the patient was taken to the ICU for resuscitation.

Prior to the planned reexploration the following day, a discussion was had with the urogynecology service. It was recommended that the patient have a formal obliterative procedure in a delayed fashion after healing from current surgical interventions and that a vaginal estrogen application be started nightly. Following this discussion, the patient underwent reexploration with stapled side-to-side functional end-to-end small bowel anastomosis and fulguration of the remnant cuff of the small bowel. She progressed well following abdominal closure and was able to be discharged home on hospital day seven.

## 3. Discussion

Vaginal cuff dehiscence with evisceration remains a rare complication of hysterectomy. Cases of dehiscence have been reported in as little as 72 hours and in very rare cases decades later [5]. The mean time to dehiscence reported in the literature varies as well given most literature is retrospective case series or case reports. Furthermore, the reported incidence varies depending on the type of hysterectomy, with open techniques reportedly having lower incidence, though this may be due to the fact that minimally invasive surgery is now the predominant form of hysterectomy performed [6]. The literature further continues to debate if the type of closure, such as single layer vs. multilayer or vaginal vs. intrabdominal, affects the risk of dehiscence with no consensus thus far reached [2].

Despite much uncertainty remaining, many risk factors have been elucidated. As in our patient, postmenopausal women appear to have increased risk due to hypoestrogenism, chronic tissue devascularization, and pelvic floor weakness [1]. Increased age, such as our patient at 79 years old, concomitant with a postmenopausal state, has also been attributed to increased risk along with vigorous Valsalva [1, 7]. However, what makes our patient scenario unique is the absence of other apparent precipitating factors and the lack of other symptoms prior to presentation. Our patient lacked antecedent events such as pelvic organ prolapse, repeated gynecologic surgery, prior intercourse, or vaginal infection. Additionally, she did not experience abdominal bleeding or discharge prior to experiencing pelvic and abdominal pain symptoms during evisceration [6].

Management of the evisceration in this case was conducted using a combined abdominal and vaginal approach. A general consensus has not been reached on the ideal repair methodology though the majority of repairs are conducted via the vaginal approach and most in open fashion though minimally invasive approaches are becoming more prevalent [3, 5]. In our case, a combined approach was necessary given the edematous nature of the eviscerated bowel requiring concerted effort between the two surgical teams to ensure adequate reduction.

In sum, vaginal dehiscence remains an infrequent, yet serious complication posthysterectomy with evisceration of the bowel a rarer and more morbid event. Here, we presented a case of evisceration several decades following index operation without typical antecedent symptoms and limited risk factors of advanced age, a postmenopausal state. The final pathology of the resected bowel demonstrated evidence of bowel ischemia without malignancy. In conclusion, this case demonstrates an enduring risk of vaginal cuff dehiscence with hysterectomy even many decades after the procedure.
